# Transcriptome-wide alternative splicing and transcript-level differential expression analysis of post-mortem Lewy body dementia brains

**DOI:** 10.1017/neu.2024.65

**Published:** 2025-02-10

**Authors:** Thomas R. Goddard, Keeley J Brookes, Kevin Morgan, Dag Aarsland, Paul Francis, Anto P. Rajkumar

**Affiliations:** 1 Institute of Mental Health, Mental Health and Clinical Neurosciences Academic Unit, Faculty of Medicine and Health Sciences, University of Nottingham, Nottingham, UK; 2 Biosciences, School of Science & Technology, Nottingham Trent University, Nottingham, UK; 3 Human Genetics, School of Life Sciences, University of Nottingham, Nottingham, UK; 4 Department of Old Age Psychiatry, Institute of Psychiatry, Psychology and Neuroscience, King’s College London, London, UK; 5 Institute for Health Research, University of Exeter Medical School, Exeter, UK; 6 Mental Health Services for Older People, Nottinghamshire Healthcare NHS foundation trust, Nottingham, UK

**Keywords:** Lewy body dementia, RNA-sequencing, alternative splicing, *TMEM18*, *GABRB3*

## Abstract

Lewy body dementias (LBD) are the second most common dementia. Several genes have been associated with LBD, but little is known about their contributions to LBD pathophysiology. Each gene may transcribe multiple RNA, and LBD brains have extensive RNA splicing dysregulation. Hence, we completed the first transcriptome-wide transcript-level differential expression analysis of post-mortem LBD brains for gaining more insights into LBD molecular pathology that are essential for facilitating discovery of novel therapeutic targets and biomarkers for LBD. We completed transcript-level quantification of next-generation RNA-sequencing data from post-mortem anterior cingulate (ACC) and dorsolateral prefrontal cortices (DLPFC) of people with pathology-verified LBD (LBD = 14; Controls = 7) using *Salmon*. We identified differentially expressed transcripts (DET) using *edgeR* and investigated their functional implications using *DAVID*. We performed transcriptome-wide alternative splicing analysis using *DRIMseq*. We identified 74 DET in ACC and 96 DET in DLPFC after Benjamini-Hochberg false discovery rate (FDR) correction (5%). There were 135 and 98 FDR-corrected alternatively spliced genes in ACC and DLPFC of LBD brains, respectively. Identified DET may contribute to LBD pathology by altering DNA repair, apoptosis, neuroplasticity, protein phosphorylation, and regulation of RNA transcription. We confirm widespread alternative splicing and absence of chronic neuroinflammation in LBD brains. Transcript-level differential expression analysis can reveal specific DET that cannot be detected by gene-level expression analyses. Therapeutic and diagnostic biomarker potential of identified DET, especially those from *TMEM18, MICB, MPO,* and *GABRB3*, warrant further investigation. Future LBD blood-based biomarker studies should prioritise measuring the identified DET in small extracellular vesicles.

## Significant outcomes

1. We present the first transcriptome-wide, transcript-level, differential expression analysis of post-mortem Lewy body dementia (LBD) brains, and we identified 169 differentially expressed transcripts (DET) and 228 alternatively spliced genes after multiple testing corrections in LBD brains.
2. Identified DET may contribute to LBD pathology by impacting DNA repair, apoptosis, protein phosphorylation, and transcription regulation.
3. Therapeutic and biomarker potential of identified DET, especially those from *TMEM18, MICB, MPO* and *GABRB3*, warrant further investigation.

## Limitations

1. Transcriptome-wide transcript-level differential expression data analysis algorithms are still evolving.
2. Functional annotations of individual RNA transcripts are limited. Our functional enrichment analysis was based on translated proteins, and it did not include the effects of identified non-coding DET.
3. Small sample size.

## Introduction

Dementia is the seventh leading cause of global mortality (Patterson, 2018). Lewy body dementias (LBD) are the second most common type of neurodegenerative dementias (Kane *et al*., 2018). They include two overlapping dementias: dementia with Lewy bodies (DLB) and Parkinson’s disease (PD) dementia (PDD) (Kane *et al*., 2018). When compared to Alzheimer’s disease (AD), people with LBD experience more rapid cognitive decline, shorter life expectancy, greater care costs, and more frequent and more severe neuropsychiatric symptoms (Svendsboe *et al*., 2016). However, LBD remain relatively under-researched. The molecular mechanisms underlying neurodegeneration in LBD are uncertain, and we do not have any disease-modifying treatment for LBD (Watts *et al*., 2023). There is no reliable biological fluid-based biomarker for LBD, and nearly 50% of people with LBD in the UK may remain misdiagnosed as other dementias (Freer, 2017). There is an urgent need for further research that advances our understanding of LBD molecular pathology. Such research is prerequisite for the discovery of novel therapeutic targets and diagnostic biomarkers that can improve clinical diagnosis and management of LBD.

We have systematically reviewed prior genetic (DNA) (Sanghvi *et al*., 2020) and gene expression (RNA) (Chowdhury and Rajkumar, 2020) studies that investigated people with LBD. Genetic studies have consistently replicated the associations between LBD and variants in *APOE*, *SNCA*, and *GBA*, and have reported genetic associations with variants in several genes including *BCL7C*, *CNTN1*, *GABRB3*, and *MAPT* (Sanghvi *et al*., 2020). DNA exert their functions by RNA transcription. Prior RNA expression and transcriptomic studies have identified many differentially expressed genes (DEG) that may contribute to LBD pathology by impacting mitochondrial dysfunction, immunosenescence, the ubiquitin-proteasome system (UPS), the autophagy lysosomal pathway (ALP), RNA-mediated gene silencing, oxidative stress and signal transduction (Chowdhury and Rajkumar, 2020, Rajkumar *et al*., 2020). However, each gene may transcribe multiple RNA transcripts with unique functions. Alternative splicing can change the proportions of expressed transcripts of a gene and consequent functions without changing the overall expression level of that gene (Wang *et al*., 2019). Gene-level DEG analyses investigate only aggregated expression levels of all transcripts from individual genes. They cannot provide information regarding differentially expressed transcripts (DET), and the extent of alternative splicing in LBD (Wang *et al*., 2019). Identifying DET and alternatively spliced isoforms has provided novel insights into the molecular pathology of AD (Raj *et al*., 2018), but there has not been any transcriptome-wide study investigating transcript-level differential expression analysis in LBD.

The only available transcriptome-wide alternative splicing investigation of LBD brains (*n* = 14) has highlighted widespread dysregulation of alternative splicing (Feleke *et al*., 2021). That study investigated only one brain region (Anterior Cingulate Cortex; ACC), did not report the most significant alternatively spliced genes, and did not conduct DET analyses. There have been candidate gene expression studies investigating alternatively spliced isoforms of *SNCA*, *SNCB*, *PRKN*, *FYN*, *APP*, *RELA*, and *ATXN2* in LBD (Beyer *et al*., 2004; Beyer *et al*., 2010; Funahashi *et al*., 2017; Low *et al*., 2021). Their findings support the importance of alternative splicing and transcript-level alterations in the molecular pathology of LBD (Beyer *et al*., 2004; Beyer *et al*., 2010; Funahashi *et al*., 2017; Low *et al*., 2021). There is a clear need to analyse transcriptome-wide data at a transcript-level resolution to provide further insight into the molecular pathology of LBD. We have previously reported transcriptome-wide DEG and consequent metabolic reprogramming in post-mortem ACC and dorsolateral prefrontal cortices (DLPFC) of people with pathology-verified LBD (Rajkumar *et al*., 2020). There is a clear requirement to analyse this next-generation RNA-Sequencing (RNA-Seq) data (Rajkumar *et al*., 2020) at transcript-level resolution. We aimed to perform the first transcriptome-wide transcript-level differential expression analysis of post-mortem LBD brains for identifying DET and alternatively spliced genes that may facilitate discovery of novel therapeutic targets and biomarkers for LBD.

## Materials and methods

### Post-mortem brain tissue

We analysed data from our prior RNA-Seq study (Rajkumar *et al*., 2020). We provide a summary of research methods here. Further details of our methodology have been published elsewhere (Rajkumar *et al*., 2020). The Brains for Dementia Research (BDR) network of brain banks (https://bdr.alzheimersresearchuk.org/researchers/), UK, provided post-mortem brain tissue, and generic ethical approval for this study (Approval 13/SC/0516 by the Oxford C Committee of the National Research Ethics Service). Post-mortem ACC (Brodmann area 24) (Pietrzak *et al*., 2016) and DLPFC (Brodmann area 9) (Bronnick *et al*., 2016) tissue samples from people with pathologically-verified LBD (*n* = 14), and from people without PD or dementia (NDC; *n* = 7) were included in this study. Supplemental Digital Content (SDC-1) presents the sample characteristics.

### RNA extraction

50 mg of tissue per brain region was obtained from each sample. They were homogenised with the T10-basic ultra-turrax and S10D-7G-KS-110 dispersing tool (Ika, USA). We extracted total RNA using the RNeasy Plus Universal Mini Kit (Qiagen, Germany).

### RNA-seq

**Figure 1.**
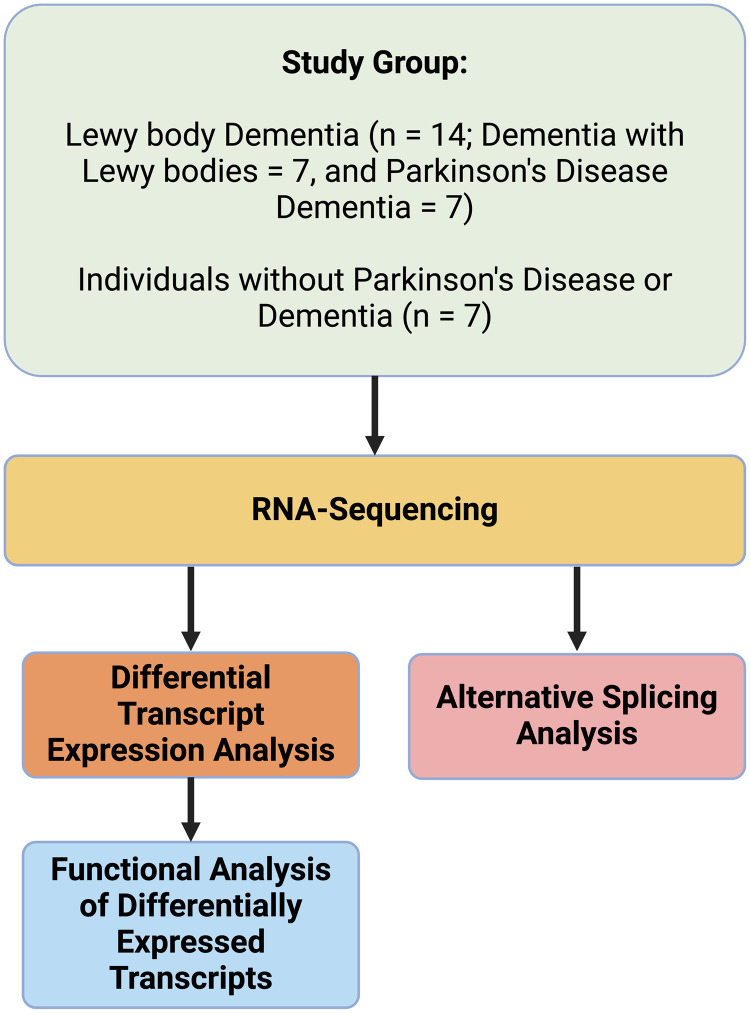
Overview of study methods.

cDNA libraries were prepared from RNA samples using TruSeq RNA sample preparation kit (Illumina, San Diego, USA). The cDNA libraries were sequenced (paired-end; 75 base pairs/read; minimum 30 million clean reads per sample) using the Illumina HiSeq-4000 (Illumina, San Diego, USA) in the Wellcome Centre for Human Genetics, Oxford, UK. Figure 1. presents an overview of study methods.

### Quantification of RNA transcripts

**Figure 2.**
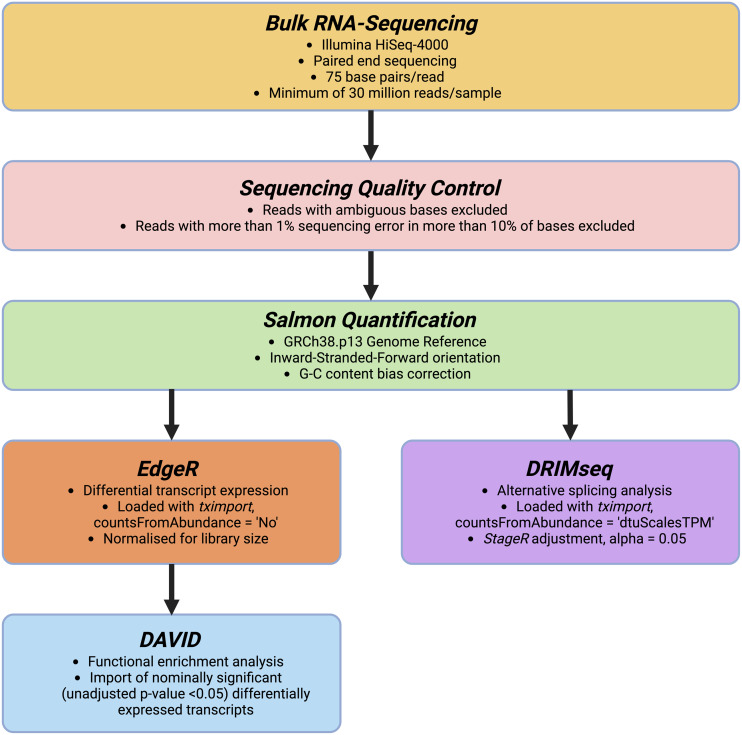
Overview of data analysis.

Figure 2. presents an overview of our data analysis pipeline. We excluded RNA-seq reads that had ambiguous bases or had more than 1% sequencing error in more than 10% bases. We quantified transcript abundance by *Salmon* (Patro *et al*., 2017), a quasi-alignment quantification tool capable of transcriptome-wide bias calculation. *Salmon* compares RNA-seq reads to a transcriptome index and performs equivalence class calculations for estimating abundance of each transcript in an RNA sample. We used the Gencode.v38 transcript fasta file (Frankish *et al*., 2023) as the index transcriptome, performed G-C content bias correction, and calculated transcript abundance. We annotated estimated transcript counts with the GRCh38.p13 genome reference.

### Transcriptome-wide DET analysis

We identified DET in LBD brains using *edgeR* (Robinson *et al*., 2010), and Benjamini-Hochberg false discovery rate (FDR) correction at 5%. *edgeR* employs negative binomial distribution for calculating differential expression from count data without degrees of freedom (df) (Robinson *et al*., 2010). The transcript count matrix was analysed for differential expression, while using a transcript length matrix as an offset within the analysis. SDC-2 presents our command scripts for supporting reproducibility.

### Alternative splicing analysis

We identified alternatively spliced genes using the transcript count matrix and *DRIMseq* (Nowicka and Robinson, 2016). *DRIMseq* employs a Dirichlet-multinomial model for comparing the relative ratio of expressed isoforms between conditions, while accounting for differential gene expression. *DRIMseq* analyses have n-1 df, while comparing two conditions. Post-hoc filtering of transcript-level tests was applied using *StageR* (Van den Berge *et al*., 2017) and an alpha of 5% for correcting the overall false discovery rate (OFDR) of *DRIMseq*. *StageR* was preferred for this correction as *DRIMseq* may exceed its FDR bounds, and application of post-hoc filtering can improve accuracy significantly (Nowicka and Robinson, 2016).

### Functional enrichment analysis

We investigated functional implications of the identified DET using the Database for Annotation, Visualization and Integrated Discovery (*DAVID)* (da Huang *et al*., 2009, Sherman *et al*., 2022). *DAVID* groups input terms into biological modules, and identifies enriched biological processes, molecular functions and Kyoto Encyclopaedia of Genes and Genomes (KEGG) pathways. We combined all nominally significant (edgeR *p* < 0.05) DET from both brain regions into a single list. We converted their Ensembl transcript IDs to UniProt Accession numbers using the biological database network (bioDBnet; https://biodbnet-abcc.ncifcrf.gov) and analysed the list of UniProt Accession numbers using *DAVID*. Such systematic functional analysis of alternatively spliced genes is not possible because of the incomplete functional annotation of the effects of alternative splicing within individual genes and the lack of a comprehensive functional annotation database for alternative splicing.

### Secondary analyses

The LBD group including both DLB and PDD samples was compared with the NDC group for identifying DET and alternatively spliced genes in LBD brains. Later, we conducted pairwise subgroup analyses comparing DLB, PDD and NDC groups using similar *edgeR* and *DRIMseq* algorithms with appropriate FDR corrections. We conducted these analyses for ACC and DLPFC separately.

## Results

196,916 and 196,360 unique RNA transcripts were expressed in ACC and DLPFC of the study samples, respectively.

### DET in LBD brains

We identified 74 FDR-corrected DET in the ACC of LBD brains. Of these, 30 were upregulated and the remaining 44 were downregulated. There were 96 FDR-corrected DET, including 31 upregulated and 65 downregulated DET, in the DLPFC of LBD brains. Table 1 presents the top 10 upregulated and downregulated DET, ranked by their FDR-corrected p-values, in ACC. Table 2 shows the top 10 upregulated and downregulated DET in DLPFC of LBD brains. Moreover, SDC-3 presents the details of all nominally significant (edgeR *p*-values < 0.05) DET in both brain regions. The ENST00000432667.5 transcript, transcribed by *TMEM18*, was significantly upregulated and expressed in all LBD DLPFC samples, but not in any NDC DLPFC sample. Furthermore, ENST00000225275.4, transcribed by the AD-associated proinflammatory *MPO*, was significantly downregulated in both regions of LBD brains after Benjamini-Hochberg FDR correction (5%).

**Table 1.** The top 10^a^ upregulated and downregulated differentially expressed transcripts in post-mortem anterior cingulate cortices of people with Lewy body dementia

Transcript ID	Gene symbol	Log fold change^b^	Adjusted *p*-value
**Up-regulated transcripts**			
ENST00000659386.1	*LINC00320*	8.997	3.21*10^-13^
ENST00000670455.1	*DLG1*	11.072	2.42*10^-7^
ENST00000651455.1	*MTR*	8.021	1.70*10^-4^
ENST00000489092.5	*AP4B1*	6.426	2.66*10^-4^
ENST00000666138.1	*LOC105374338*	9.257	4.39*10^-4^
ENST00000560117.1	*AQR*	5.833	4.88*10^-4^
ENST00000662184.1	*MYO6*	8.922	1.27*10^-3^
ENST00000486530.1	*HPS3*	7.665	1.44*10^-3^
ENST00000416996.1	*RP11-120J1.1*	6.863	2.66*10^-3^
ENST00000375405.7	*SCN1A*	11.461	4.37*10^-3^
**Down-regulated transcripts**			
ENST00000587169.5	*CIRBP*	−5.979	4.31*10^-4^
ENST00000681594.1	*OAS3*	−4.854	5.79*10^-4^
ENST00000225275.4	*MPO*	−4.939	1.06*10^-3^
ENST00000397281.6	*REPIN1*	−8.608	1.21*10^-3^
ENST00000509063.5	*ALB*	−5.429	1.94*10^-3^
ENST00000299667.9	*ZNF3*	−7.981	2.66*10^-3^
ENST00000360057.7	*TPK1*	−7.219	4.37*10^-3^
ENST00000372997.3	*NUDT13*	−7.586	4.37*10^-3^
ENST00000558709.1	*SCG3*	−4.523	4.37*10^-3^
ENST00000677370.1	*NPEPPS*	−7.855	4.37*10^-3^

^a^Top differentially expressed transcripts as ranked by their adjusted *p*-values (Benjamini-Hochberg false discovery rate correction at 5%); ^b^Base of 2; Analysis was completed using *edgeR*. *edgeR* estimates dispersion with a negative binomial distribution and calculates differential expression from count data. Transcript length was used as an offset and this analysis was performed without df.

**Table 2.** The top 10^a^ upregulated and downregulated differentially expressed transcripts in post-mortem dorsolateral prefrontal cortices of people with Lewy body dementia

Transcript ID	Gene symbol	Log fold change^b^	Adjusted *p*-value
**Up-regulated transcripts**			
ENST00000432667.5	*TMEM18*	9.054	2.94*10^-31^
ENST00000546331.5	*RERG*	7.968	6.18*10^-9^
ENST00000380324.8	*USP3*	10.194	7.64*10^-7^
ENST00000678630.1	*KPNA4*	10.651	7.64*10^-7^
ENST00000417122.7	*ITGB1*	9.048	9.80*10^-4^
ENST00000523108.5	*MGAT4B*	7.650	2.50*10^-3^
ENST00000492129.5	*PRPSAP2*	8.945	2.54*10^-3^
ENST00000672031.1	*ACAT1*	8.598	2.62*10^-3^
ENST00000414665.5	*MBOAT7*	6.585	3.41*10^-3^
ENST00000571378.5	*SRRM2*	9.415	4.16*10^-3^
**Down-regulated transcripts**			
ENST00000355654.6	*TMEM18*	−8.907	9.62*10^-13^
ENST00000403059.8	*PVR*	−8.600	4.73*10^-12^
ENST00000533410.5	*RNH1*	−8.926	4.89*10^-12^
ENST00000339746.9	*ANKRD6*	−9.134	1.18*10^-10^
ENST00000439495.6	*ALS2*	−8.690	8.47*10^-6^
ENST00000426960.6	*ARL6IP4*	−8.517	1.61*10^-5^
ENST00000519415.6	*OXR1*	−9.002	1.61*10^-5^
ENST00000639435.1	*BIVM-ERCC5*	−8.971	2.28*10^-5^
ENST00000242066.9	*ETV1*	−7.783	1.35*10^-4^
ENST00000465416.5	*CCNY*	−7.335	1.60*10^-4^

^a^Top differentially expressed transcripts as ranked by their adjusted *p*-values (Benjamini-Hochberg false discovery rate correction at 5%); ^b^Base of 2; Analysis was completed using *edgeR*. *edgeR* estimates dispersion with a negative binomial distribution and calculates differential expression from count data. Transcript length was used as an offset and this analysis was performed without df.

### DET in DLB and PDD brains

SDC-3 presents the details of all nominally significant DET that were identified by DET subgroup analyses, comparing the transcriptomes of both brain regions of the DLB samples with corresponding NDC and PDD samples. When compared to the NDC samples, there were 129 and 121 FDR-corrected DET in the ACC and DLPFC of DLB samples, respectively. While comparing the PDD samples, we identified 143 and 114 FDR-corrected DET in the ACC and DLPFC of DLB samples, respectively. ENST00000262327.9, transcribed by DNA ligase *LIG3*, was the top DET with the least FDR-corrected p-value (8.30*10^-23^) in the ACC of DLB brains. ENST00000682046.1, transcribed by DNA-binding *THAP12*, was the top DET (FDR-corrected *p*-value = 3.32*10^-10^) in the DLPFC of DLB brains, when compared to PDD brains. Moreover, when compared to the NDC samples, we identified 130 and 156 FDR-corrected DET in the ACC and DLPFC of PDD samples, respectively.

### Alternative splicing in LBD brains

We detected 135 significantly alternatively spliced genes in the ACC of LBD brains after OFDR correction (5%). There were 98 significantly alternatively spliced genes including *TMEM18*, *GOLGA2*, *CTTN*, *ARHGEF4* and *SHC2* in the DLPFC of LBD brains after similar OFDR correction. Table 3 presents the top 20 alternatively spliced genes, ranked by their OFDR-corrected *p*-values, in both brain regions. *SGSH*, *NAV2*, *ZC3H7A*, *FAM76A* and *SURF1* were the top five alternatively spliced genes in ACC of LBD brains. SDC-4 presents further results from the *DRIMseq* transcriptome-wide alternative splicing analysis of both LBD brain regions. Four genes, *MYL6*, *CTTN*, *ING3,* and *LOC105374338*, were significantly alternatively spliced after OFDR correction in both LBD brain regions.

**Table 3.** The top 20^a^ alternatively spliced genes within anterior cingulate and dorsolateral prefrontal cortices of people with Lewy body dementia

ACC^b^		DLPFC^c^	
Gene symbol	Adjusted *p*-value^d^	Gene symbol	Adjusted *p*-value^d^
*SGSH*	1.11*10^-9^	*TMEM18*	3.81*10^-11^
*NAV2*	9.42*10^-7^	*GOLGA2*	2.06*10^-7^
*ZC3H7A*	1.45*10^-6^	*CTTN*	6.14*10^-6^
*FAM76A*	1.07*10^-5^	*ARHGEF4*	9.93*10^-6^
*SURF1*	3.28*10^-5^	*SHC2*	2.31*10^-5^
*LOC105374338*	3.28*10^-5^	*PRSS51*	5.24*10^-5^
*UBE2Q1*	6.91*10^-5^	*TBC1D17*	5.69*10^-5^
*AATK*	6.91*10^-5^	*EFTUD2*	5.69*10^-5^
*MIR124-1HG*	1.86*10^-4^	*NPDC1*	8.61*10^-5^
*HPS3*	2.13*10^-4^	*DLG4*	1.22*10^-4^
*TPGS2*	3.05*10^-4^	*LOC105374338*	1.30*10^-4^
*IQGAP1*	3.07*10^-4^	*CDC42SE1*	1.92*10^-4^
*MTR*	3.40*10^-4^	*NDUFS8*	2.79*10^-4^
*AQP4*	3.90*10^-4^	*PRMT1*	2.79*10^-4^
*AP4B1*	6.33*10^-4^	*PLCG1*	4.09*10^-4^
*LINC01001*	6.33*10^-4^	*KPNA4*	4.09*10^-4^
*NHP2*	6.48*10^-4^	*EIF4G1*	4.09*10^-4^
*BRIX1*	6.48*10^-4^	*RERG*	4.26*10^-4^
*CDK5RAP2*	6.99*10^-4^	*POLR3A*	4.95*10^-4^
*RFTN1*	7.17*10^-4^	*CFAP20*	6.72*10^-4^

^a^Top 20 alternatively spliced genes as ranked by their adjusted *p*-values; ^b^Anterior cingulate cortex; ^c^Dorsolateral prefrontal cortex; ^d^*P*-values adjusted with *StageR*, alpha = 5%. Alternative splicing analysis was performed with *DRIMSeq*. *DRIMSeq* utilises a Dirichlet-multinomial model to compare the relative ratio of expressed isoforms between conditions. Degrees of freedom was set as q-1.

### Alternative splicing of TMEM18 and MICB

**Figure 3.**
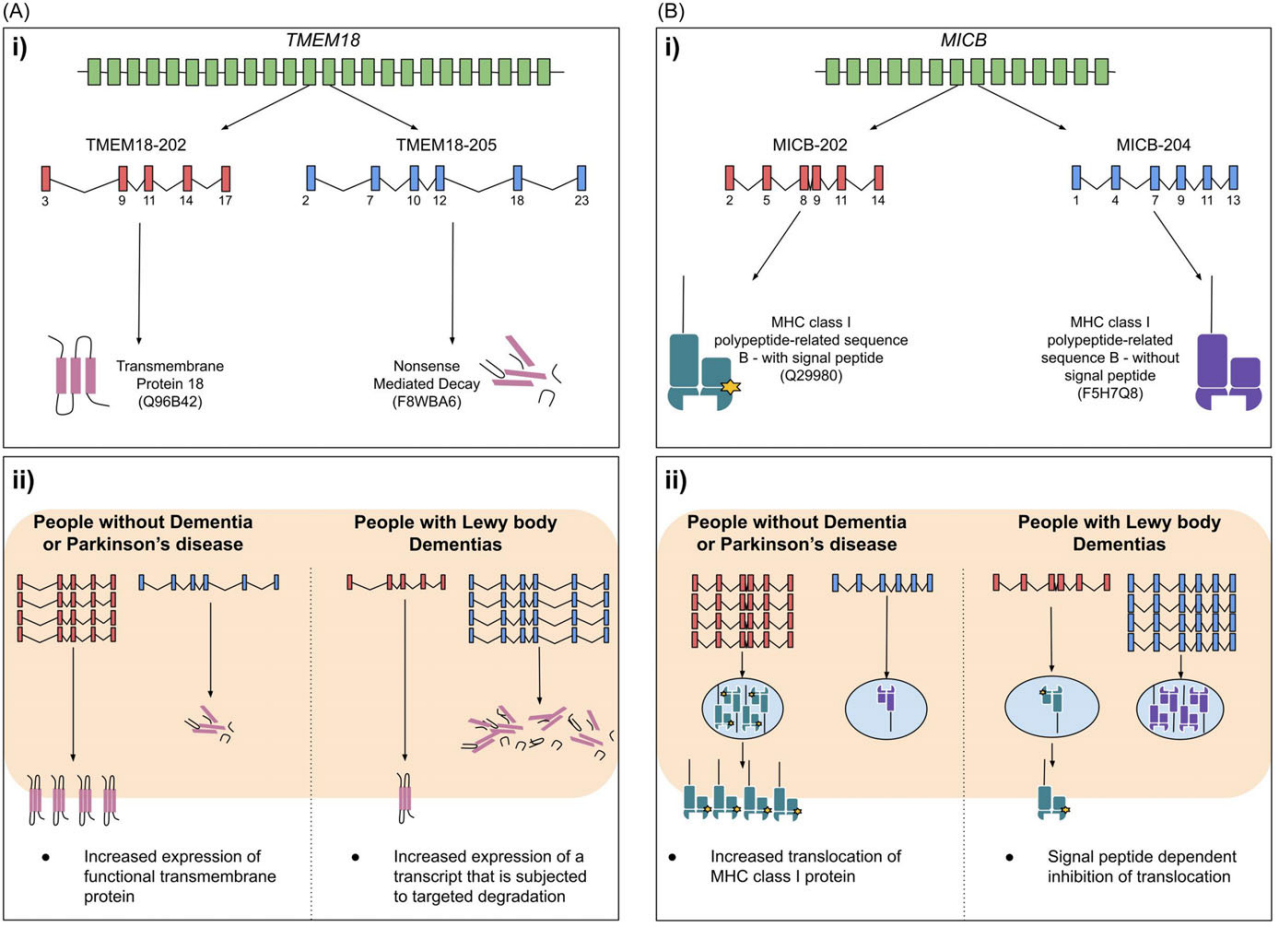
Alternative splicing of the *TMEM18* and *MICB* genes in post-mortem dorsolateral prefrontal cortices of the Lewy body dementia (LBD) brains. Figure-3-A-i shows the functional implications of two transcripts of *TMEM18* gene. TMEM18-202 translates into transmembrane protein 18 (Q96B42) and TMEM18-205 leads to nonsense mediated decay product (F8WBA6). Figure-3-A-ii shows the potential effects of alternative splicing of *TMEM18* gene in dorsolateral prefrontal cortices of the LBD brains. TMEM18-202 was significantly upregulated in people without dementia or Parkinson’s disease (NDC group), when compared to people with LBD (18.64% of *TMEM18* transcripts Vs 0.00%). TMEM18-205 was significantly upregulated in people with LBD when compared to the NDC group (22.08% of *TMEM18* transcripts Vs. 0.00%). Such alternative splicing is likely to lead to increased nonsense mediated decay of translated protein in LBD brains. Figure-3-B-i shows the functional implications of two transcripts of *MICB*. MICB-202 and MICB-204 translate into an MHC class 1 polypeptide-related sequence with (Q29980) or without (F5H7Q8) signal peptide, respectively. Figure-3-B-ii shows the potential effects of alternative splicing of *MICB* gene in dorsolateral prefrontal cortices of the LBD brains. MICB-202 was significantly upregulated in the NDC group, when compared to people with LBD (47.70% of *MICB* transcripts Vs. 4.47%). MICB-204 was significantly upregulated in people with LBD when compared to the NDC group (26.96% of *MICB* transcripts Vs 0.00%). Such alternative splicing is likely to lead to impaired translocation of the MHC class 1 protein.

Figure 3. presents an overview of possible mechanisms by which alternative splicing of *TMEM18* and of *MICB* may contribute to LBD pathology. *TMEM18* is the top alternatively spliced gene in the DLPFC of LBD brains (Table-3; SDC-4). It also transcribes the top upregulated DET and the top downregulated DET in the same region (Table-2; SDC-3). The top downregulated transcript, TMEM18-202, translates into functional Transmembrane Protein 18 (UniProt:Q96B42; TMM18), and the top upregulated transcript, TMEM18-205, leads to a nonsense mediated decay product (UniProt:F8WBA6). TMEM18-202 represented 18.64% of all *TMEM18* transcripts within the NDC brains, and it was not detected in any LBD brains. TMEM18-205 represented 22.08% of all *TMEM18* transcripts within the LBD brains, and it was not detected in any NDC brain. Similarly, *MICB* is an OFDR-corrected alternatively spliced gene (OFDR-corrected *p*-value = 1.85*10^-3^) in the DLPFC of LBD brains. *MICB* transcribes MICB-202 and MICB-204 that translate into an MHC class 1 polypeptide-related sequence with (UniProt:Q29980) or without (UniProt:F5H7Q8) a signal peptide, respectively. In LBD brains, 26.96% of all *MICB* transcripts were MICB-204, whilst none of the NDC brains expressed MICB-204. Conversely, MICB-202 represented 47.70% of all *MICB* transcripts within the NDC group, and it only represented 4.47% of transcripts within the LBD group.

### Alternative splicing in DLB and PDD brains:

SDC-4 presents *DRIMseq* transcriptome-wide alternative splicing subgroup analyses, comparing the transcriptomes of both DLB brain regions with corresponding NDC and PDD samples. When compared to the NDC group, there were 49 and 84 OFDR-corrected significantly alternatively spliced genes in the ACC and DLPFC of DLB brains, respectively. When compared to the PDD group, we found 96 and 100 OFDR-corrected alternatively spliced genes in the ACC and DLPFC of DLB brains, respectively. *SGSH*, associated with a lysosomal storage disease, was the top alternatively spliced gene with the lowest OFDR-corrected p-value (6.06*10^-7^) in the ACC of DLB brains. *TMEM18* was one of the top five alternatively spliced genes (OFDR-corrected *p*-value = 8.88*10^-6^) in the DLPFC of DLB brains, when compared to NDC brains. Furthermore, when compared to the NDC group, there were 133 and 110 OFDR-corrected alternatively spliced genes in the ACC and DLPFC of PDD brains, respectively.

### Functional enrichment analysis of identified DET

We analysed the functional implications of all unique proteins that are translated by the identified nominally significant (edgeR *p* < 0.05) DET from both brain regions of LBD brains. Table-4 presents the top 10 enriched biological processes and molecular functions, ranked by their FDR-corrected *p*-values, in LBD brains. SDC-5 presents all FDR-corrected significantly enriched biological processes, molecular functions and KEGG pathways in LBD, DLB and PDD brains, when compared to NDC brains. It also presents the FDR-corrected enriched biological processes, molecular functions and KEGG pathways in DLB brains, when compared to PDD brains. Proteins, translated by the identified LBD brain DET, were significantly enriched for 70 biological processes, 46 molecular functions and 63 KEGG pathways after FDR correction. They may contribute to LBD pathology by impacting DNA repair, signal transduction, apoptosis, protein phosphorylation, regulation of RNA transcription, vesicle-mediated transport, regulation of cell cycle, histone deacetylation, UPS, ALP, and the Wnt signalling pathway. Similarly, proteins, translated by the identified DLB brain DET, are likely to contribute to DLB pathology by affecting cell cycle, apoptosis, protein phosphorylation, proteolysis, regulation of RNA transcription, vesicle-mediated transport, ALP, and signal transduction.

**Table 4.** Functional enrichment analysis^a^ of identified differentially expressed transcripts^b^ in post-mortem brains of people with Lewy body dementia

Gene ontology term	Adjusted *p*-value^c^
**Biological process**	
DNA repair	8.80*10^-9^
Protein phosphorylation	1.70*10^-7^
Positive regulation of transcription, DNA-templated	2.50*10^-7^
Telomere organization	4.40*10^-7^
Regulation of cell cycle	2.30*10^-6^
Regulation of catalytic activity	2.30*10^-6^
Cell cycle	4.60*10^-6^
Cell division	2.70*10^-5^
Intracellular signal transduction	2.70*10^-5^
Response to drug	4.80*10^-5^
**Molecular function**	
Protein binding	3.40*10^-69^
ATP binding	5.60*10^-22^
Identical protein binding	8.90*10^-16^
Cadherin binding	4.50*10^-8^
Protein kinase activity	1.40*10^-7^
Small GTPase binding	1.40*10^-7^
Ubiquitin protein ligase binding	7.90*10^-7^
Kinase activity	2.90*10^-6^
RNA binding	2.90*10^-6^
DNA binding	7.10*10^-6^

^a^Top ten biological process and molecular function terms as ranked by their adjusted *p*-values; ^b^Nominally significant differentially expressed transcripts in either anterior cingulate or dorsolateral prefrontal cortices; ^c^*p*-values adjusted by Benjamini-Hochberg false discovery rate correction at 5%; Analysis was completed using DAVID: https://david.ncifcrf.gov/. DAIVD functional analysis utilises a modified Fisher’s exact test without df to create an EASE score for gene enrichment analysis.

## Discussion

This is the first transcriptome-wide study that analysed transcript-level differential expression and investigated alternatively spliced genes at transcript-level resolution in post-mortem LBD brains. It has identified 169 novel DET and 228 alternatively spliced genes in post-mortem LBD brains after appropriate FDR corrections. It has found specific transcripts, TMEM-205 and MICB-204, that were expressed exclusively in LBD samples. It confirms widespread alternative splicing in ACC of LBD brains (Feleke *et al*., 2021), and presents the first transcriptome-wide transcript-level differential expression analysis of LBD prefrontal cortices. However, the limitations of this study include its small sample size, lack of replication experiments such as quantitative real-time PCR, and the lack of single cell RNA-seq data. Moreover, functional enrichment analysis was based on translated proteins and that did not include the effects of identified non-coding DET. The current knowledge on the functions of individual non-coding RNA transcripts is very limited. When there are better methods for predicting the targets of identified non-coding DET, and a database for transcript-level functional knowledge of non-coding RNA, further functional enrichment analysis can be performed by including the identified non-coding DET.

Prior gene-level analysis of these RNA-seq data has identified 12 FDR-corrected DEG (Rajkumar *et al*., 2020). This transcriptome-wide DET analysis demonstrates the limitations of gene-level DEG analysis and the extent of novel insights that can be gained by transcript-level analyses. *MPO* was the top DEG, ranked by FDR-corrected p-values, in the ACC and it was one of the top three FDR-corrected DEG in the DLPFC of these LBD brains (Rajkumar *et al*., 2020). An *MPO* transcript, ENST00000225275.4 (MPO-201; Uniprot:P05164) was the only FDR-corrected DET that was significantly differentially expressed in both LBD brain regions. Downregulation of MPO-201 is likely to cause reduced translation of Myeloperoxidase protein. Myeloperoxidase plays an important role in oxidative stress and neuroinflammation, and it mediates proteolysis. *MPO* variants have been associated with AD (Reynolds *et al*., 1999). Myeloperoxidase co-localizes with amyloid plaques in AD brains, and its plasma levels were reportedly higher in people with AD (Tzikas *et al*., 2014). Chronic neuroinflammation is well established in AD brains but several lines of evidence indicate the absence of chronic neuroinflammation in LBD, especially in DLB (Rajkumar *et al*., 2020; Amin *et al*., 2020; Rajkumar *et al*., 2021). The downregulation of MPO-201 may contribute to the differences in neuroinflammation in LBD brains, and functional consequences of this downregulated DET warrant further research. Moreover, *GABRB3* encodes a subunit of gamma-aminobutyric acid (GABA) type-A receptor. A *GABRB3* transcript, ENST00000638099.1 (GABRB3-223; Uniprot:A0A1B0GVW3), was significantly downregulated in the ACC of LBD brains (SDC-3). This finding, and previously reported genome-wide significant association of a *GABRB3* variant (rs1426210) with LBD (Sanghvi *et al*., 2020), support the importance of GABAergic dysfunction in LBD pathology.

Designing oligonucleotide probes for measuring specific RNA transcripts is relatively easier than measuring overall expression levels of genes with multiple transcripts through targeted gene expression assays. Post-mortem DLB brain DEG have been found significantly enriched among the DEG identified in serum small extracellular vesicles (SEV) of people living with DLB (Rajkumar *et al*., 2021). Statistically significant differential expression of many DEG in DLB brains could be measured in the blood-based SEV of people living with DLB (Rajkumar *et al*., 2021). Hence, the identified FDR-corrected DET, especially those expressed exclusively in LBD brains, not only advance our understanding of LBD molecular pathology, but also warrant further investigation of their biomarker potential in people living with LBD.

*TMEM18* was the top OFDR-corrected alternatively spliced gene in the DLPFC of LBD brains. *TMEM18* encodes TMM18 protein. It regulates adipogenesis, gene silencing, and neuronal migration, as well as promoting neuroplasticity (Jurvansuu and Goldman, 2011, Jurvansuu *et al*., 2008; Luck *et al*., 2020). The TMEM18-205 transcript was found only in LBD samples. Alternative splicing of *TMEM18* and significant upregulation of TMEM18-205 are likely to reduce TMM18 expression by increasing nonsense mediated decay in LBD brains. Impaired transcription repression due to reduced TMM18 levels may lead to upregulation of other pathogenic transcripts. Such gene regulatory changes, and the consequent dysfunctional molecular networks in LBD brains, should be investigated further. Moreover, TMM18 interacts with two proteins, RETR3 (Uniprot:Q86VR2) and REEP4 (Uniprot:Q9H6H4) (Luck *et al*., 2020), both of which are associated with Endoplasmic Reticulum (ER) morphology (Kumar *et al*., 2019). ER dysfunction and consequent increased activation of the unfolded protein response may contribute to LBD pathology (Baek *et al*., 2016). Furthermore, reduced expression of TMM18 is likely to impair migration of neural stem cells and neuroplasticity (Jurvansuu *et al*., 2008). Such impaired neuroplasticity may lead to neurodegeneration and cognitive decline in LBD (Toricelli *et al*., 2021).

Identified alternative splicing of *MICB* may lead to a greater proportion of MHC proteins without a signal peptide. This may lead to signal peptide-dependent inhibition of protein translocation, and reduced expression of the functional MHC class-I protein (Powers and Fruh, 2008) in the DLPFC of LBD brains. *MICB* was significantly alternatively spliced only in DLB brains and not in PDD brains. *MICB* shares location (6p21.33) and function with a previously reported DEG, *HLA-B*, in LBD brains (Feleke *et al*., 2021; Cunningham *et al*., 2022). The MICB protein (Uniprot:Q29980) is stress induced and it activates the cytolytic response of natural killer cells (Baranwal and Mehra, 2017). Upregulation of MICB and consequent neuroinflammation have been reported in AD brains (Garranzo-Asensio *et al*., 2018). Alternative splicing of *MICB* may lead to reduced cytolytic response of natural killer cells in the DLPFC of DLB brains, and this may contribute to the absence of chronic neuroinflammation in DLB brains (Rajkumar *et al*., 2020; Amin *et al*., 2020).

Prior candidate gene expression studies that investigated the alternatively spliced isoforms of *SNCA*, *SNCB*, and *APP* have reported significant associations with LBD (Beyer *et al*., 2004; Beyer *et al*., 2010; Funahashi *et al*., 2017; Low *et al*., 2021), and those alternative splicing findings have not been replicated by our transcriptome-wide analysis. However, it replicated significant alternative splicing of another previously reported alternatively spliced gene, *FYN*, in LBD brains (Beyer *et al*., 2004). *FYN* is associated with cell survival and immune response (Low *et al*., 2021). Prior studies have suggested that *FYN* alternative splicing is driven by increased expression of isoforms that are primarily expressed in T-cells (FynT) (Low *et al*., 2021). We did not find statistically significant differential expression of FynT transcripts, and alternative splicing of *FYN* was likely due to upregulation of FYN-218 (ENST00000517419.5). This transcript was an FDR-corrected DET in the DLPFC of DLB brains, and little is known about its function. Moreover, identified alternatively spliced genes include *GOLGA2*, *ABCB9* and *RHBDD1*, which may contribute to protein aggregation in LBD brains (Baek *et al*., 2016).

Identified protein coding DET in LBD brains were significantly enriched for several biological processes and molecular pathways that are relevant to α-synuclein aggregation, Lewy body formation, and neurodegeneration. α-synuclein modulates DNA repair, and DNA repair deficits contribute to neurodegeneration in LBD (Schaser *et al*., 2019). Identified DET are also likely to impact Ubiquitin protein ligase binding, which is essential for the post-translational modification of proteins and the removal of misfolded protein aggregates (Zhang *et al*., 2019). Changes in Ubiquitin ligase binding and UPS dysfunction may impact the post-translational modification of α-synuclein, and could promote Lewy body formation (Zhang *et al*., 2019). Further research investigating the contributions of the identified DET towards dysfunctional apoptosis, protein phosphorylation, RNA transcription, vesicle-mediated transport, the UPS, the ALP, and Wnt signalling in LBD may facilitate discovery of novel therapeutic targets. When compared to the functional understanding of genes and proteins, the current understanding of functions of individual RNA transcripts is limited. Fortunately, transcript-level functional knowledge is quickly expanding, and this will help functional interpretation of DET analysis in the future. The DET identified within this study should be verified with independent biological replicates and be the subject of future investigations focussing on their dysfunctional molecular networks and functional consequences. Further functional insight can be gathered by multi-omic analysis that combines transcript-level differential expression analysis with genomic, proteomic, and/or epigenetic data. Moreover, investigating the potential of the identified DET in mitigating α-synuclein aggregation in appropriate in vitro and in vivo models will enhance their clinical translation into potential novel therapeutic targets for LBD and other synucleinopathies.

We cannot overemphasise the need for adequately powered transcriptome-wide transcript-level studies investigating LBD brains and blood-based SEV of people living with LBD. If such studies include comparisons with AD or other dementia, they can facilitate discovery of diagnostic biomarkers for LBD. Plasma SEV RNA can be novel blood-based diagnostic biomarkers for DLB (Rajkumar *et al*., 2021). A prior study has demonstrated statistically significant overlap between post-mortem DLB brain DEG and the DEG, identified in blood-based SEV from people living with DLB (Rajkumar *et al*., 2021). Parallelly measuring multiple RNA biomarkers together may improve their diagnostic accuracy, and the identified DET in LBD set the stage for developing potential multiplex RNA diagnostic biomarker assays for LBD. Moreover, unlike DNA polymorphisms, RNA expression levels remain dynamic during disease progression. Hence, investigating blood-based expression levels of the identified DET at various clinical stages of LBD may aid early diagnosis of LBD and may lead to the discovery of novel prognostic biomarkers for LBD.

## Supporting information

Goddard et al. supplementary material 1 Goddard et al. supplementary material

Goddard et al. supplementary material 2 Goddard et al. supplementary material

Goddard et al. supplementary material 3 Goddard et al. supplementary material

Goddard et al. supplementary material 4 Goddard et al. supplementary material

Goddard et al. supplementary material 5 Goddard et al. supplementary material

## Data Availability

Additional data including raw RNA-seq data files, transcript count matrix files and transcript level alternative splicing proportions are available upon reasonable request to the corresponding author.

## Supplementary material

The supplementary material for this article can be found at https://doi.org/10.1017/neu.2024.65.
