# Supplementary material for: Transcriptome-wide alternative splicing and transcript-level differential expression analysis of post-mortem Lewy body dementia brains
Source: Acta Neuropsychiatr. 2025 Feb 10;37:e9. doi: 10.1017/neu.2024.65 (PMC13130329; doi:10.1017/neu.2024.65)
Supplement: Goddard et al. supplementary material 1 — Goddard et al. supplementary material [file S0924270824000656sup001.docx]

**SDC-1**: Sample characteristics

| **Participant** | **Diagnosis** | **Age in years** | **Gender** | **PMI** | **ACC** | **DLPFC** | **A score** | **B score** | **C score** |
| --- | --- | --- | --- | --- | --- | --- | --- | --- | --- |
| 1 | DLB | 79 | Male | 115.00 | Yes | Yes | NA | NA | NA |
| 2 | DLB | 66 | Male | 23.00 | No | Yes | NA | 3 | NA |
| 3 | DLB | 89 | Male | 40.00 | Yes | Yes | NA | 1 | 0 |
| 4 | DLB | 81 | Male | 8.50 | Yes | Yes | 2 | 3 | 2 |
| 5 | DLB | 94 | Male | 32.25 | Yes | Yes | NA | 2 | NA |
| 6 | DLB | 80 | Male | 89.35 | Yes | Yes | 1 | 2 | 0 |
| 7 | DLB | 81 | Male | 32.50 | Yes | Yes | 2 | 3 | 2 |
| 8 | PDD | 81 | Male | 18.10 | Yes | Yes | 3 | 1 | 2 |
| 9 | PDD | 82 | Male | 47.14 | Yes | Yes | 0 | 2 | 0 |
| 10 | PDD | 92 | Male | 41.35 | Yes | Yes | 3 | 2 | 1 |
| 11 | PDD | 81 | Male | 38.00 | Yes | Yes | 1 | 1 | 1 |
| 12 | PDD | 86 | Female | 78.10 | Yes | Yes | 3 | 1 | 2 |
| 13 | PDD | 80 | Male | 77.00 | Yes | Yes | 2 | 1 | 1 |
| 14 | PDD | 73 | Male | 30.30 | Yes | Yes | 3 | 1 | 2 |
| 15 | NDC | 92 | Male | 34.25 | Yes | Yes | 1 | 1 | 1 |
| 16 | NDC | 89 | Male | 54.50 | Yes | Yes | 1 | 1 | NA |
| 17 | NDC | 81 | Male | 35.75 | Yes | Yes | 1 | 2 | NA |
| 18 | NDC | 84 | Male | 79.10 | Yes | Yes | 0 | 0 | 0 |
| 19 | NDC | 84 | Male | 76.50 | Yes | Yes | 1 | 2 | 1 |
| 20 | NDC | 87 | Male | 39.25 | Yes | No | 1 | 1 | 0 |
| 21 | NDC | 83 | Male | 39.45 | Yes | Yes | NA | 2 | 1 |

DLB: Dementia with Lewy bodies; PDD: Parkinson’s disease dementia; NDC: Controls without cognitive impairment; PMI: post-mortem interval in hours; ACC: Availability of anterior cingulate cortex tissue; DLPFC: Availability of dorsolateral prefrontal cortex tissue; A score: β-amyloid plaque score (A0: No β-amyloid plaques, A1: Thal phases 1 or 2, A2: Thal phase 3, A3: Thal phases 4 or 5); B score: Neurofibrillary tangle stage (B0: No Neurofibrillary tangles, B1: Braak stage I or II, B2: Braak stage III or IV, B3: Braak stage V or VI); C score: The Consortium to Establish a Registry for Alzheimer's disease (CERAD) neuritic plaque score (C0: No neuritic plaques, C1: CERAD score sparse, C2: CERAD score moderate, C3: CERAD score frequent); NA: Score was not available.
