# Supplementary material for: Transcriptome-wide alternative splicing and transcript-level differential expression analysis of post-mortem Lewy body dementia brains
Source: Acta Neuropsychiatr. 2025 Feb 10;37:e9. doi: 10.1017/neu.2024.65 (PMC13130329; doi:10.1017/neu.2024.65)
Supplement: Goddard et al. supplementary material 2 — Goddard et al. supplementary material [file S0924270824000656sup002.docx]

**Salmon Quantification:**

conda install salmon

conda create -n salmon salmon

conda activate salmon

cd ...

#Creating salmon index

salmon index --gencode -t gencode.v38.transcripts.fa -i gencode.v38_salmon-1.9.0

cd ...

salmon quant -p 6 -i gencode.v38_salmon-1.9.0 -l A \ --gcBias -o sample -1 *SampleNumberForwardRead*.fastq -2 *SampleNumberReverseRead*.fastq

cd ...

mv quant.sf *SampleNumber*quant.sf

**Importing into R:**

library(devtools)

library(GREP2)

setwd("...")

library(rnaseqDTU)

samps <- read.csv(file.path("...", "..."))

samps$condition <- factor(samps$condition)

table(samps$condition)

files <- file.path("...",samps$sample_id)

names(files)<- samps$sample_id

head(files)

library(tximport)

txi <- tximport(files, type="salmon", txOut=TRUE,

countsFromAbundance="scaledTPM")

txi.t <- tximport(files, type="salmon", txIn=TRUE, txOut=TRUE, countsFromAbundance="no")

txi.g <- tximport(files, type="salmon", tx2gene=txdf[,2:1])

cts <- txi$counts

cts <- cts[rowSums(cts) > 0,]

head(cts)

library(GenomicFeatures)

setwd("...")

gtf <- "gencode.v38.chr_patch_hapl_scaff.annotation.gtf"

txdb.filename <- "gencode.v38.annotation.sqlite"

txdb <- makeTxDbFromGFF(gtf)

saveDb(txdb, txdb.filename)

txdb <- loadDb(txdb.filename)

txdf <- select(txdb, keys(txdb, "GENEID"), "TXNAME", "GENEID")

tab <- table(txdf$GENEID)

txdf$ntx <- tab[match(txdf$GENEID, names(tab))]

range(colSums(cts)/1e6)

write.table(colSums(cts)/1e6,"ACC_LBDNDC_Counts.text")

head(txdf)

all(rownames(cts) %in% txdf$TXNAME)

txdf <- txdf[match(rownames(cts),txdf$TXNAME),]

all(rownames(cts) == txdf$TXNAME)

counts <- data.frame(gene_id=txdf$GENEID, feature_id=txdf$TXNAME, cts)

**Alternative Splicing Analysis:**

library(DRIMSeq)

d<-dmDSdata(counts=counts,samples=samps)

methods(class=class(d))

counts(d[1,])[,1:4]

table(table(counts(d)$gene_id))

design_full <- model.matrix(~condition, data=DRIMSeq::samples(d))

colnames(design_full)

set.seed(1)

system.time({

d <- dmPrecision(d, design=design_full)

d <- dmFit(d, design=design_full)

d <- dmTest(d, coef="condition2")})

res <- DRIMSeq::results(d)

head(res)

res.txp <- DRIMSeq::results(d, level="feature")

head(res.txp)

no.na <- function(x) ifelse(is.na(x), 1, x)

res$pvalue <- no.na(res$pvalue)

res.txp$pvalue <- no.na(res.txp$pvalue)

idx <- which(res$adj_pvalue < 0.05)[1]

res[idx,]

plotProportions(d, res$gene_id[idx], "condition")

nrow(res)

nrow(res.txp)

pScreen <- res$pvalue

strp <- function(x) substr(x,1,15)

names(pScreen) <- strp(res$gene_id)

pConfirmation <- matrix(res.txp$pvalue, ncol=1)

rownames(pConfirmation) <- strp(res.txp$feature_id)

tx2gene <- res.txp[,c("feature_id", "gene_id")]

for (i in 1:2) tx2gene[,i] <- strp(tx2gene[,i])

library(stageR)

stageRObj <- stageRTx(pScreen=pScreen, pConfirmation=pConfirmation,

pScreenAdjusted=FALSE, tx2gene=tx2gene)

stageRObj <- stageWiseAdjustment(stageRObj, method="dtu", alpha=0.05)

suppressWarnings({

drim.padj <- getAdjustedPValues(stageRObj, order=FALSE,

onlySignificantGenes=FALSE)})

head(drim.padj)

res.txp.filt <- DRIMSeq::results(d, level="feature")

smallProportionSD <- function(d, filter=0.1) {

cts <- as.matrix(subset(counts(d), select=-c(gene_id, feature_id)))

gene.cts <- rowsum(cts, counts(d)$gene_id)

total.cts <- gene.cts[match(counts(d)$gene_id, rownames(gene.cts)),]

props <- cts/total.cts

propSD <- sqrt(rowVars(props))

propSD < filter

filt <- smallProportionSD(d)

res.txp.filt$pvalue[filt] <- 1

res.txp.filt$adj_pvalue[filt] <- 1

res.txp.filt$tranSTRP <- strp(res.txp.filt$feature_id)

res.txp.filt.gene <- merge(drim.padj, res.txp.filt,by.x="txID",by.y="tranSTRP")

drim.group.prop<-proportions(d)

drim.group.gene.prop <- merge(res.txp.filt.gene, drim.group.prop,by.x="feature_id",by.y="feature_id")

drim.group.prop.counts <- merge(drim.group.gene.prop,txi.t$counts,by.x="feature_id",by.y="row.names")

write.table(drim.group.prop.counts,"...",col.names=T,row.names=F,quote=F,sep="\t")

**Differential Transcript Expression Analysis:**

library(edgeR)

cts.t <- txi.t$counts

normMat <- txi.t$length

normMat <- normMat / exp(rowMeans(log(normMat)))

o <- log(calcNormFactors(cts.t/normMat)) + log(colSums(cts.t/normMat))

y <- DGEList(cts.t)

y <- scaleOffset(y, t(t(log(normMat)) + o))

y <- estimateDisp(y, design_full)

fit <- glmFit(y, design_full)

lrt <- glmLRT(fit)

tt <- topTags(lrt, n=nrow(y), sort="none")[[1]]

tt.t.counts <- merge(tt,txi.t$counts,by.x="row.names",by.y="row.names")

write.table(tt.t.counts,"...",col.names=T,row.names=F,quote=F,sep="\t")
